# Filling the Gaps for Feeding Difficulties in Neonates With Hypoxic-Ischemic Encephalopathy

**DOI:** 10.7759/cureus.28564

**Published:** 2022-08-29

**Authors:** Ishani Arora, Heena Bhandekar, Ashwini Lakra, Mahaveer S Lakra, Sandhya S Khadse

**Affiliations:** 1 Department of Paediatrics, Datta Meghe Medical College and Shalinitai Meghe Hospital and Research Center, Nagpur, IND; 2 Department of Paediatrics, Jawaharlal Nehru Medical College, Datta Meghe Institute of Medical Sciences, Wardha, IND

**Keywords:** swallowing impairment, speech and swallowing therapy, mechanisms, hpoxic ischemic encephalopathy, feeding difficulties

## Abstract

Hypoxic-ischemic encephalopathy (HIE) in neonates poses long-term feeding difficulties and abnormalities of swallowing, the sequel of which is growth impairment. Such infants are also at risk of impaired self-feeding in the grown-up stage along with other motor and tone abnormalities leading to malnutrition and multiple aspiration pneumonia episodes. The lack of evidence-based and pragmatic feeding strategies in such neonates is because of varied unrecognized symptoms and lacking validated diagnostic approaches. This article approaches evidence related to the pathophysiologic basis of feeding difficulties in neonates with HIE as well as standardizing measures and techniques to improve the feeding abilities of such babies and, in turn, their long-term development. The present review provides a scaffold for putting importance on this less taken care issue of feeding problems and emphasizes that more objective and evidence-based studies are required to be added to the literature for early interventions and management of this issue so that caregivers and neonatologists are not misguided by crude subjective opinions.

## Introduction and background

Hypoxic-ischemic encephalopathy (HIE) is a dreaded complication due to birth asphyxia, where oxygen supply to the brain is compromised antepartum, intrapartum, or immediately postpartum. It is a problem of great concern worldwide and causes approximately 900,000 deaths every year and is one of the main causes of neonatal death and morbidity [1]. The approximate incidence is around three to five per 1000 live births in developed countries, including moderate or severe HIE in up to one per 1000 live births [2,3]. But the condition is worse in developing countries, as in India, the incidence of HIE reaches up to 10-15 per 1000 live births [4]. According to a prospective study in a tertiary care center in an African country, the authors found an 11% incidence of HIE, out of which 39% constituted moderate HIE and almost 10% as severe HIE [5]. Most neonates encountering perinatal brain injury die due to complications of asphyxia in early neonatal life, specifically with severe hypoxic injury, but the survivors exhibit various long-term neuromotor complications such as cerebral palsy (CP), intellectual disability, epilepsy, or motor abnormalities like tone abnormalities, involuntary movements, and feeding difficulties. Of all the problems, feeding difficulties are the most pernicious and are seen in >50% of severely affected cases [6,7]. Identifying and understanding this less taken care problem of feeding difficulties in such neonates is essential. In India, the healthcare system is overburdened, and tertiary care neonatal intensive care units (NICUs) are loaded with HIE babies; hence, their rehabilitation of feeding difficulties is missed during discharge and follow-up. Creating awareness of feeding difficulties in these neonates during the early phase of life is very important. And more important is creating appropriate measures and new scientific strategies for better feeding in these neonates, which will create evidence-based measures with new scientific methods that will foster a better way to feed HIE neonates leading to a better quality of life, improving parental satisfaction, and decreasing the overall economic burden in the society at large. Lack of awareness and proper knowledge on how to deal with these feeding difficulties leads to deleterious long-term individual and social impacts. The discordance between pathophysiologic basis and realistic feeding techniques remains an enigma basically due to a lack of well-studied diagnostic procedures and consensuses in management. A dedicated and objectified evaluation to understand basic pathophysiology and varied symptoms in neonates with HIE is needed for superlatively individualized therapies.

## Review

The burden of feeding difficulties in HIE neonates

Neonates with HIE have significant feeding difficulties, which further lead to failure to thrive, impaired growth and development, and increased morbidity and mortality. In a follow-up study in Rwanda, high-risk infants were followed up for six months, and almost one-third had poor growth up to six months of age and 16% had moderate acute malnutrition, reflecting the chronic detrimental effect of feeding difficulties [8]. Martinez-Biarge et al. found in their study that around 65% of perinatal asphyxia survivors suffered feeding impairment and about 50% required long-term nasogastric tube placement. They found that basal ganglia and thalamic injury and mesencephalic lesions were related to feeding difficulties and the intensity of basal ganglia and pontine insult were directly associated with the need for gastrostomy and can be correlated from early MRI scans [9]. In a study by Fung et al., the authors concluded that malnutrition in CP, a consequence of HIE, and feeding problems were very common due to poor oral-motor function and were very distressing for parents and eventually led to the child’s poor health and nutrition status [10]. Similarly, Hou et al. studied oral motor dysfunctions and feeding problems in children with CP leading to malnutrition and concluded that a thorough evaluation for feeding problems should be done as early as possible in such cases so that interventions can be initiated timely to improve nutrition status and quality of life [11].

Symptoms and mechanisms behind feeding difficulties in HIE

For swallowing to happen safely, complete coordination is required in the oropharyngeal, upper esophageal sphincter (UES), esophagus, lower esophageal sphincter (LES), and upper airway pathways along with their coordinated movements. Intact normal swallowing mechanisms are essential for handling oral secretions and proper passage of bolus into the esophagus with a coordinated mechanism for airway protection, but aberrant reflexes cause many distressing symptoms in newborns with HIE. Hence, the mechanisms causing feeding difficulties in neonates with HIE are abnormal UES functioning in the form of its increased tone and abnormal contractile reflex, decreased LES basal tone, increased relaxation reflex, and also poorly coordinated esophageal peristaltic responses. Oropharyngeal muscular tonicity problems cause abnormal alignment and coordination causing feeding difficulties in neonates with HIE [12]. Table 1 shows multiple phases of feeding and their features.

**Table 1:** Phases of feeding in newborns and features pertaining to HIE HIE: hypoxic-ischemic encephalopathy; UES: upper esophageal sphincter; LES: lower esophageal sphincter.

Phases of feeding in newborns and features pertaining to HIE		
Phase	Features	Abnormality in HIE
Primary	Alert and active state, rooting reflex, non-nutritive sucking	Sleepy and dull state, poor rooting reflex
Oral	Sucking and swallowing in a 1:1 ratio	Increased oral secretions, shallower sucking, majority of short and single sucking bursts
Pharyngeal	Swallowing, deglutition apnea, reflexive pharyngeal swallow, UES contractile and relaxation reflexes, LES relaxation, pharyngo-glottal closing reflex	Aberrant deglutition, decreased deglutition frequency and duration, impaired provoked pharynx reflexes, and smooth muscle contractility
Esophageal	UES contractile reflex, primary and secondary peristalsis, UES relaxation reflex, esophageal deglutition reflex, secondary peristalsis	Increased tonicity of skeletal muscle components of UES, prolonged and poorly coordinated peristalsis

A case study by Krüger et al. described breastfeeding and swallowing difficulties in a single neonate, assessing clinically and using a video-fluoroscopic swallow study, and concluded that breastfeeding difficulties were present due to the oral stage of dysphagia and were exacerbated by poor state regulation [13]. Further, Krüger et al. in their study identified various symptoms of swallowing difficulties in neonates with HIE. They found that increased sleepiness during feeding, excess oral secretions, coughing, and nasal flare were the most frequent symptoms. In addition to this, poor rooting, shallower latching onto the breast, and the majority of single bursts of sucks in neonates with HIE were found as compared to a more alert and active state in normal term neonates [14]. Short sucking bursts lead to difficulty in swallowing and ineffective breastfeeding. In an experimental study, Gulati et al. demonstrated that the central generation of swallowing reflex, frequency of swallowing and its duration, UES and LES tone, and activity during provoked pharynx test were abnormal in HIE neonates as compared to normal controls [15]. Assessing pharyngoesophageal protective reflexes in HIE neonates by Jensen et al. yielded that as compared to controls, HIE neonates had increased skeletal muscle tone, impaired pharyngeal provocation-induced reflexes, and smooth muscle contractility. The contractility was also increasingly abnormal with the maturation of age and these mechanisms may be responsible for inadequate handling of secretions, movement of ascending reflux, and oropharyngeal bolus in HIE infants [16]. Hill and Jadcherla, in their study of esophageal peristalsis in HIE infants, proved that mechano-distention of the esophagus in HIE infants results in uncoordinated and delayed peristaltic responses leading to aerodigestive dysregulation, and sensorimotor reflex of the aerodigestive tract is also altered in infants with HIE [17]. Not only in HIE but also in surgical cases of congenital heart disease (CHD), Malkar and Jadcherla studied the neuromotor mechanisms of pharyngoesophageal motility in dysphagic infants with CHD and found excitatory responses in cholinergic vagus neuron complexes leading to an abnormality in the recruitment of pharyngo-upper esophageal contractile response, pharyngeal swallow reflexes, and esophageal body peristalsis [18].

Regardless of the problems, neonates with HIE in NICU have several difficulties in starting and sustaining feeds due to associated comorbidities like necrotizing enterocolitis (NEC) due to mesenteric ischemia and cellular death. Moreover, infants with early neurological insult have complex initial courses like ventilation, continuous positive airway pressure (CPAP), humidified high-flow nasal cannula (HHFNC), or other respiratory support and also they go through various procedures leading to prolonged NICU stay and are separated from their mothers. The combination of such factors causes a delay in the attainment of composite rooting, sucking, and swallowing milestones in newborns with HIE.

Diagnosis

In neonates with HIE, neuromotor coordination at the esophageal level is abnormal and during further development, this aberrancy increases, leading to dysfunctional neuronal reflexes and motor responses leading to oromotor dyspraxia. This in turn produces such motor responses that are not appropriate for safe and effective oral feeding. Hence, abnormalities in neuromotor discoordination and tone leading to feeding difficulties should be detected early like the increased tone of the UES and its prolonged contractility, which can be an early therapeutic target. Focused anatomic and physiologic assessment is sufficient via thorough clinical assessment, hence observation of a complete oral feeding session is a good strategy for knowing the alertness level of the baby, body tone, latching, swallowing, pauses in between sucking and swallowing, respiratory effort, and drooling, if present. But sometimes multiple diagnostic studies are required to look for anatomic with the functional aspects of the feeding issues. However, judicious and individual base choice of diagnostic methods is very important to properly interpret the results and get applicability of the results, specifically in resource-limited scenarios like in India. Table 2 shows various diagnostic methods available along with their advantages and limitations.

**Table 2:** Diagnostic tests and their characteristics NICU: neonatal intensive care unit.

Test	Advantage	Disadvantage
Video-fluoroscopic swallow study (VSS)	Assess anatomy, bolus movement detection during swallowing, and laryngeal aspiration detection	Requires transport of neonate, uses barium, radiation use, nonuniformity in testing methods and interpretations. Cost and availability limitations
Fiberoptic endoscopic evaluation of swallowing (FEES)	Assess anatomy, movements of structures, and dynamic swallowing. Can be carried out bedside in NICU. Therapeutic in guiding individual feeding plan	Cost and availability limitations. Operator expertise required
Pharyngoesophageal manometry	Pharyngeal swallowing pressures and esophageal body peristalsis at rest and on provocation. A better predictor of feeding outcomes as compared to VSS	Less commonly available. Clinical correlation needed

Video-fluoroscopic swallow study (VSS) is a commonly used and available modality to see the oropharyngeal and esophageal anatomy related to swallowing for safe feeding. Other modalities used can be upper gastrointestinal fluoroscopy, fiberoptic endoscopy, and pharyngoesophageal manometry. Diagnostics tests for predicted issues should be performed as early as possible to detect particular pathology in individual infants and should be followed by individually targeted preventive and curative measures [19]. The VSS is commonly used, but the disadvantage to the VSS is that it uses radiation and barium dye and neonates have to be transported to radiology, also it has limited options for seeing different positions. In developing countries, the most important are cost and availability issues, along with that, its indications and interpretations are not standardized for newborns. Another approach is fiberoptic endoscopic evaluation of swallowing (FEES), in which a flexible endoscope is passed through the nasal cavity into the pharynx to see the anatomy, dynamic movement of structures, swallowing function, and effect of therapeutic interventions. Reynolds et al. concluded that FEES is a safe alternative to VSS as it has the advantage of being carried out in the NICU at the bedside, and is not only diagnostic but also therapeutic with the ability to replicate precise feeding scenarios. This can assist in providing a safe feeding plan further [20]. Also, in a study by Jadcherla et al., by evaluating neonatal dysphagia, they studied 20 neonates having abnormal VSS, and assessed swallow reflexes using pharyngoesophageal motility via using micromanometers catheter and pneumo-hydraulic water perfusion system. They found that disease characteristics or VSS results do not correlate much with feeding outcomes; however, manometry can be a better predictor for feeding outcomes than VSS for identifying newborns likely to benefit from various feeding intervention programs [21]. Once identifying a feeding issue, a collaborative multidisciplinary team-based approach is needed in the initial phase of care, that is at the time of preparing the baby for discharge from the NICU.

The promising solution to the problem for improved outcomes

Preventing feeding difficulties is the best alternative, but HIE is invariably associated with feeding issues and for prevention strategies to prevent birth asphyxia, the whole healthcare system is required to be geared up. Although in newborns with hypoxic injury, timely intervention and prevention of severe outcomes are essential for better outcomes. Appropriately timed and properly provided hypothermic therapy during the early phase has a proven protective role and later improves swallowing and aerodigestive mechanisms leading to better oral feeding skills at one year [14]. An individualized approach for each neonate with HIE with feeding difficulties is needed. Early assessment and identification are very much essential and simultaneously early stimulation-regulation exercises of swallowing reflexes should be a part of care in neonates with the sequel of HIE [22]. Proper communication with parents, care providers, and allied healthcare professionals is necessary for making adequate feeding strategies and implementing them. Bapat et al. proved by using a “simplified, individualized, milestone targeted, pragmatic, longitudinal, and educational (SIMPLE)” feeding strategy that optimizing a particular process and taking protocol-based and simple innovative quality improvement measures for neonatal feedings in a tertiary care center NICU can make the difference. They concluded that implementing a standardized feeding strategy in NICU minimizes inter-caregiver variability and accelerates the attainment of feeding milestones [23,24]. In a study in rural Rwanda to promote breastfeeding and overcome difficulties of feeding in neonates with HIE and low birth weight (LBW), various interventions were done like training of allied clinical staff (nurses and other supporting staff from the pediatrics and obstetrics side) by speech and language therapists (SLTs) for supporting breastfeeding in infants with feeding difficulties. They concluded that neonates with prematurity, LBW, and/or HIE require individualized special support to promote early breastfeeding and overcome the feeding difficulties in the early phase [8]. Neonates with HIE have various tone abnormalities, as discussed earlier in this review, which limit the progression of their feeding milestones compared with normal term neonates; hence, they need an individualized approach for the management of feeding in them. A study by Jadcherla et al. has promised great hope in dealing with feeding difficulties in neonates with HIE. This study included 100 neonates who were analyzed at birth and manometric studies of pharynx and esophagus along with swallow studies were done to evaluate the structure and physiology of the aerodigestive system for each neonate, and they received an individualized feeding strategy, which was multidisciplinary, individualized, and evidence-based. In total, 51% of such infants were feeding successfully at the time of discharge and 84% at one year of age as compared to only 10 that were feeding orally at discharge and 42% at one year of age prior to this study program [25]. The early involvement of the SLTs in the treatment of feeding difficulties in neonates and infants is very much required. An effective and early involvement of SLTs within the neonatal team leads to early identification of at-risk neonates, which will lead to necessary individualized therapy to start early [26]. Early interventions like sensory stimulations and teaching oral motor exercises to mothers or caregivers and sensitizing and educating them regarding the importance of taking dedicated actions in the early phase is very much essential. In a retrospective study by Sharp et al., studying children dependent on enteral feedings, the authors found increased caloric intake and weight gain, 33% weaning from tubes, and better caregiver satisfaction by using interventions like behavioral training, caregiver training, and oral-motor exercise [27]. Multiple similar trials have been conducted by Arslan et al., Kaviyani Baghbadorani et al., and Sığan et al. using various methodologies to improve feeding like proper positioning, oral motor exercises, sensory stimulations, training, and sensitizing caregivers individually [28-30]. All these studies have provided positive results that things can be made better with dedicated efforts and proper application of science as needed. It is very important to note that all the trials done for dealing with feeding difficulties and improving nutrition and health status utilized more than one modality, again emphasizing the multimodality approach and multidisciplinary team-based approach.

## Conclusions

HIE is a common and serious problem in the neonatal period, specifically in resource-limited low- and middle-income countries. Most neonates develop long-term feeding complications that are more difficult to manage. Hypoxic brain injury affects swallowing coordination leading to tone abnormalities in the oropharyngeal-esophageal muscles and peristalsis abnormalities, consequently leading to ineffective feeding and other debilitating symptoms. Judicious and individual use of available diagnostic procedures such as VSS and FESS (which can be therapeutic also sometimes) should be done for early diagnosis. Further, providing early, individualized multidisciplinary management is the mainstay. Each NICU, especially tertiary level, should be equipped with a team of allied professionals like speech-language therapists, lactational counselors, and occupational therapists to prepare parent-neonate pairs for safe discharge. Pathophysiology-based individualized feeding strategy is required to be inculcated in the management of such neonates in achieving successful feeding and, in turn, long-term outcomes.
